# Unraveling the Intricacies of the Seminal Microbiome and Its Impact on Human Fertility

**DOI:** 10.3390/biology13030150

**Published:** 2024-02-27

**Authors:** Celia Corral-Vazquez, Joan Blanco, Zaida Sarrate, Ester Anton

**Affiliations:** Genetics of Male Fertility Group, Unitat de Biologia Cel·lular (Facultat de Biociències), Universitat Autònoma de Barcelona, 08193 Cerdanyola del Vallès, Spain; ccorral@researchmar.net (C.C.-V.); joan.blanco@uab.cat (J.B.); zaida.sarrate@uab.cat (Z.S.)

**Keywords:** microbiome, semen, sperm, fertility, human, bacteria, viruses

## Abstract

**Simple Summary:**

This review summarizes the main findings about the human seminal microbiome regarding its biological origins, microbial populations, and relationship with fertility and health, as well as the factors that may affect its composition and the potential sources of data variability.

**Abstract:**

Although the microbial communities from seminal fluid were an unexplored field some decades ago, their characteristics and potential roles are gradually coming to light. Therefore, a complex and specific microbiome population with commensal niches and fluctuating species has started to be revealed. In fact, certain clusters of bacteria have been associated with fertility and health, while the outgrowth of several species is potentially correlated with infertility indicators. This constitutes a compelling reason for outlining the external elements that may induce changes in the seminal microbiome composition, like lifestyle factors, gut microbiota, pathologies, prebiotics, and probiotics. In this review, we summarize the main findings about seminal microbiome, its origins and composition, its relationship with fertility, health, and influence factors, while reminding readers of the limitations and advantages introduced from technical variabilities during the experimental procedures.

## 1. Introduction

Mammalian bodies contain microbial communities that inhabit commensally in diverse tissues. These niches include bacteria, viruses, and fungi and are known as microbiota. The microbiota and the host establish a mutually beneficial relationship, with the host providing favorable habitat conditions for microorganisms, and the microbiota supports good development of the immune and metabolic system [1]. In particular, the human microbiome is restricted to exterior tissues and invaginations like skin, mucosa, gut, or genital tract [2] and is mainly composed of *Firmicutes* and *Bacteroidetes* bacterial phyla (92%) [3]. Nevertheless, the niches of microbiota found in the human body are not a stable population but a dynamic set of communities that change over the years and are influenced by factors like the environment, diet, lifestyle, and diseases [4,5].

Although the microbiome of the main human niches has been thoroughly profiled during the last few decades, only a small proportion of studies were dedicated to characterizing the seminal microbiota. The evolution of the microbial profiling technologies over the years have allowed us to define the main populations of bacteria and viruses that inhabit the seminal fluid and their variations due to external factors like sexual life, diseases, or infertility. Many authors have focused on this last topic and found the association between the over-representation of certain species and specific effects related with fertility disorders. 

The potential role of semen microbiome in fertility has become a field of special interest, given that infertility constitutes a growing problem that affects 8–12% of couples worldwide, with male-specific factors contributing to 40–50% of those cases [6]. Knowing the mechanisms that determine the influence of seminal microbiota over fertility opens new doors to developing novel approaches and treatments. In this sense, the potential use of beneficial bacterial species through the intake of prebiotics and probiotics is starting to display an interesting potential.

Nevertheless, the study of microbiome still presents important and ubiquitous limitations. There is a lack of consensus in the methodology, and the diversification of sample processing, profiling technology, and data analysis give rise to variability and biases in the results. To overcome these handicaps, several initiatives have emerged to suggest guidelines and protocols in order to standardize the arbitrary aspects of the methodologies and reveal the biases and potential contaminations associated with certain techniques.

In this review, we recapitulate the main aspects of the microbiota of the human seminal fluid. From the first studies to the recent publications, we discuss its composition and characteristics, the clusters associated with health and fertility, and the species correlated with fertility disorders. The influence of external elements over the microbial composition, the potential of probiotics, and the technical considerations of microbiome seminal studies are also summarized.

## 2. Origin of the Seminal Microbiome and General Composition

Originally, it was believed that the microbiota detected in seminal fluid was a footprint of previous or current infections in the urogenital tract and a potential reflection of inadequate cellular immune responses [7,8]. With the application of novel technologies, the idea of a commensal community of seminal microorganisms has arisen since bacterial and virus populations have been detected in samples from patients who are healthy, infected, fertile, or infertile [9,10]. In fact, the slightly basic pH and molecular composition of semen compose a suitable habitat for microorganisms [11]. 

Therefore, the composition of seminal microbiota results from the contribution of diverse zones and fluids of the urogenital tract, like urine, the urethra, and the coronal sulcus [11]. Also, a contribution of the gut microbiota is possible since there is a strong interaction between the gut microbiome and the regulation of testicular functions, known as the gut–testes axis [12].The testicular and epididymal contribution to the seminal microbiome community has been suggested since an alteration of semen microbiota has been identified after vasectomy [13]. Also, there is an overlap between the microorganism profiles of seminal fluid and urine/testicular samples [13,14,15,16,17]. Nevertheless, these repertoires are only partially shared, so semen harbor species not displayed in first-catch urine samples [18], suggesting a specific microbiome niche. 

From the first characterization studies of the human seminal microbiota [19,20], many authors have researched its microbial imprint. Although the results obtained through the years have been heterogeneous and variable, possibly due to differences in methodology and in the selection of the study cohort (see Section 5), a common pattern starts to be elucidated.

The most comprehensively characterized microorganisms in human semen are bacteria. Some specific genera are especially frequent among profiling studies, like *Staphylococcus*, *Streptococcus*, *Lactobacillus*, *Corynebacterium*, *Prevotella*, *Escherichia*, *Anaerococcus*, *Enterococcus*, *Finegoldia*, *Peptinophilus*, *Vogesella* [7,8,10,16,21,22,23,24,25,26,27,28,29,30,31,32,33,34,35,36,37,38,39], and also *Gardnerella*, *Campylobacter*, *Ureaplasma*, *Haemophilus*, *Klebsiella*, and *Pseudomonas* [7,10,20,21,22,24,25,27,30,33,34,35,36,38,39]. Other genera like *Acinetobacter*, *Cutibacterium*, *Porphyromonas*, *Chlamydia*, *Bacillus*, *Burkholderia*, *Morganella*, *Pelomonas*, and *Proteus* were identified [10,20,22,29,30,33,34,35,36,38,39].

The nucleic acid footprint of viruses have also been detected in the human seminal fluid, probably persisting after infections that have been transmitted to the genital tract, especially in cases of viremia [40]. They can be found as free virus particles, attached to molecules on the outside of spermatozoa or inside them. In fact, apart from mature spermatozoa, they can also infect their precursor cells and seminal immune cells [41]. Signs of several viruses from the *Flaviviridae* family have been persistently detected in semen samples, especially Zika virus (ZKV) [39,40,42]. Different species from the *Herpesviridae* family, like herpesvirus and cytomegalovirus (HCMV) [39,40,43,44,45,46], and from the *Papillomaviridae* family [43,44,45,47,48] are also frequent, as well as *Parvoviridae* like adeno-associated viruses (AAV) [40,44]. The human immunodeficiency virus (HIV) is one of the most cited virus regarding seminal microbiota [49]. Several studies have detected SARS-CoV-2 in semen after COVID-19 infection [50,51]. 

## 3. The Semen Microbiome in Fertility Disorders

As mentioned before, the seminal microbiota is involved in the maintenance and regulation of homeostasis and health. Specifically, its potential role in fertility is becoming clear. It is known that infections and inflammatory reactions in the male genital tract are the cause of around 6–10% of infertility cases [49]. In fact, many studies have discovered that fertile and infertile populations displayed different bacterial cohorts in the seminal fluid. Data obtained so far point out that infertility implies a higher richness and diversity of microbial elements, with an increase in alpha diversity (relative to the number of different taxa) [9,13]. The dysbiosis and abundant detection of microorganisms have been related to different indicators of poor fertility status, like seminal ROS, sperm DNA fragmentation, and the disruption of Protamine 1/Protamine 2 (P1/P2) ratio [16,37,52,53]. It is known that abnormal sperm P1/P2 ratios are related with higher DNA fragmentation and also with sperm parameters and fertilization capacity [52]. Moreover, the high presence of specific bacterial genera has been related to fertility disorders. Some examples are *Cutibacterium*, *Rhodopseudomonas* [21], *Aerococcus* [13], Varibaculum, *Escherichia* [9], *Mycoplasma*, *Ureaplasma* [54], or *Chlamydia*. In particular, *U. urealyticum*, *U. parvum*, and *M. hominis*, which inhabit the male urethra and contaminate semen during ejaculation, are known to play an etiologic role in genital infections and infertility, although their mechanisms have not been discovered yet [54,55]. Furthermore, infection by *C. trachomatis* has been associated with the induction of sperm apoptosis and impacting fertilizing ability [14,56].

**Figure 1:**
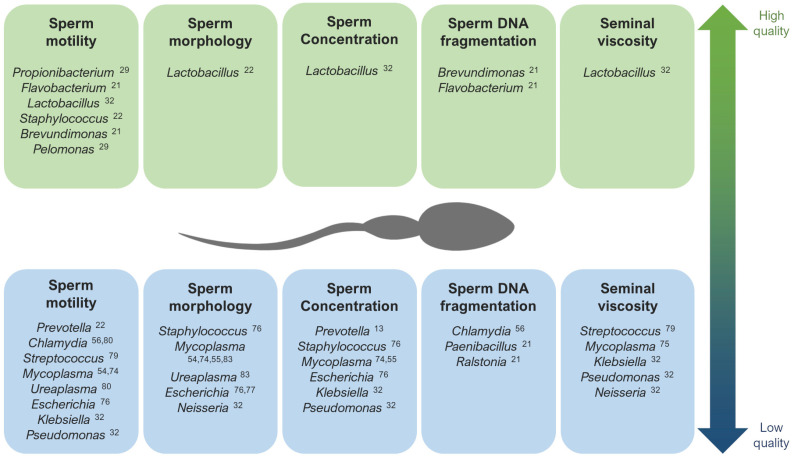
The main genera that have been described as potentially associated with high quality (**up**) and low quality (**down**) seminal parameters [13,21,22,29,32,54,55,56,74,75,76,77,79,80,83].

When studying alterations in the seminal microbiome that might affect fertility, the correlation between the presence of diverse bacteria and viruses and abnormalities in sperm parameters has been assessed [7,9,25,37,52] (Figure 1). A general low quality of semen has been associated with a high prevalence of *Gardnerella*, *Prevotella* [13,22,25,57], *Anaerococcus* [10], *Enterococcus*, and *Streptococcus* [58]. Moreover, many authors have found low quality semen parameters associated with infections caused by HIV, human papillomavirus (HPV), herpes simplex virus 1 and 2 (HSV), hepatitis B (HBV), and hepatitis C virus (HCV) [59,60,61,62,63,64,65,66,67,68,69,70,71,72,73]. Seminal infections by *Mycoplasma* have been associated with low sperm motility [54,74], abnormal morphology, low sperm concentration [55,74], and higher viscosity [75]. The specie *Neisseria gonorrhoeae* was found abundant in patients with seminal hyperviscosity and low rates of normal sperm morphology, count, and motility [32], although some authors have observed a positive relationship between this bacteria and sperm motility [34]. *Pseudomonas* has also shown an abundance in samples with high viscosity and low sperm count and motility [32], although there are contradictions in other studies [13]. *Escherichia* is another example of bacteria associated with low sperm motility, concentration, and normal form rates [76,77]. In fact, some studies suggest that *Escherichia coli* produces sperm immobilization, which affects their morphology and acrosomal function [77], and the soluble factors secreted by this bacteria inhibit the mitochondrial membrane potential, motility, and vitality of human spermatozoa [78]. 

Regarding other species suspected to affect specific sperm parameters, infections with the bacteria *Streptococcus*, *Klebsiella*, *Ureaplasma parvum*, and *Chlamydia trachomatis*, as well as ZIKV and AAV, have been associated with low rates of progressive and non-progressive motility of sperm cells [32,56,79,80,81,82]. In general, the lipopolysaccharide contained in the cell walls of gram-negative bacteria (like *Bacteroidia*, *Sphingobacteria*, *Proteobacteria*, or *Alphaproteobacteria*) are suspected to disrupt sperm motility [28]. Low rates of normal morphology in sperm have been observed in samples with the abundance of *Staphylococcus aureus*, *Ureaplasma urealyiticum*, HCMV, and human polyomavirus 2 (JCPyV) [34,76,83,84,85], and low sperm counts have been associated with the presence of *Staphylococcus*, *Haemophilus*, *Klebsiella*, *Chlamydia trachomatis*, and ZIKV [25,32,56,74,76,82]. Patients with azoospermia have shown more abundance of *Mycoplasma* and *Ureaplasma* [27], as well as an increase in *Bacteroidetes*, *Firmicutes* [86], *Proteobacteria*, and *Actinobacteria* [87].

Contrarily, *Lactobacillus* tends to predominate in the semen microbiota of healthy and fertile men with good quality semen parameters [22,31,32,57,78,88]. *Brevundimonas*, *Staphylococcus*, *Flavovacterium*, and *Pelomonas* have also been detected in patients with good seminal indicators, like high rates of sperm motility and low DNA fragmentation [21,22,27,29] (Figure 1). 

Lastly, when studying the microbiome composition of semen in relation with pregnancy rate, men involved in spontaneous pregnancy loss displayed the presence of *Porphyromonas* and *Campylobacter* [89], *S. aureus* and *E. coli* [90], as well as a high viral diversity [43]. Contrarily, high rates of success in assisted reproduction techniques (ART) has been associated with *Acinerobacter*, *Lactobacillus Jesenia*, and *Faecalibacterium* [27,89].

## 4. Influence of External Factors over Sperm Microbiome Composition

**Figure 2:**
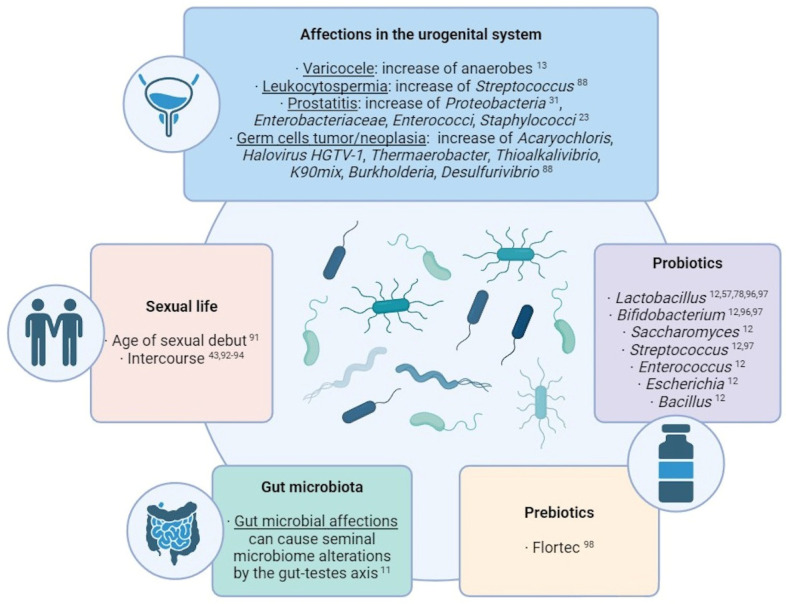
Summary of the main external factors with an influence over the seminal microbiome composition [11,12,13,23,31,43,57,78,88,91,92,93,94,96,97,98].

Several studies have made direct associations between external factors and the transformation of seminal microbial composition (Figure 2). Commensal microbiota can suffer alterations due to the influence of external factors. One of the most studied cases in the human body is the gut microbiota: many studies have revealed an influence of factors like lifestyle, age, ethnicity, geographical location, body mass index, food, diseases, and treatments over the composition of the microbiota residing in the guts [11]. Taking into account the microbial connection through the gut–testes axis, these factors affecting the gut microbiome may also produce alterations in the seminal microbiota. 

The effect of sexual life over the seminal microbiome has been documented over the years. Mändar et al. identified that sexual debut implies an increase in the diversity and concentration of seminal bacteria [91] and that the species identified in the seminal fluid (and vagina) change after intercourse [92], which has been supported by other studies [93]. Therefore, a common core of bacteria and viruses is shared between seminal fluid and vagina [43,94]. This transmission appears to be a stochastic and passive and has been suggested to display a role in reinforcing the sexual health and in facilitating sperm functionality and fertilization [95]. Nevertheless, diseases of one partner can also produce deleterious alterations in the other partner’s microbiota (e.g. reduction in *L. crispatus* and predominance of *G. vaginalis* in vaginal samples after intercourse with a partner with genital tract inflammation [92,93]).

In this sense, alterations in the urogenital system can also produce modifications in its own semen microbiota. For example, an over-representation of anaerobes has been observed in seminal samples of patients with varicocele [13], and Streptococcus-enriched microbial communities are associated with leukocytospermia [88]. In cases of prostatitis, a wide species richness and a higher proportion of *Proteobacteria* was observed [31], while the growth of *Enterobacteriaceae*, *Enterococci*, and *Staphylococcus* was observed in the semen of chronic prostatitis syndrome patients compared with healthy men [23]. Regarding testicular germ cell tumors and germ cell neoplasia, high levels of *Acaryochloris marina*, *Halovirus HGTV-1*, *Thermaerobacter marianensis*, *Thioalkalivibrio* sp. *K90mix*, *Burkholderia* sp. *YI23*, and *Desulfurivibrio alkaliphilus* have been detected in the seminal plasma of the affected patients [88].

To counteract these effects, the use of probiotics and prebiotics for improving the seminal microbiome and the fertility condition is being widely considered and studied. Prebiotics are products that promote the growth of beneficial microorganisms, while probiotics themselves contain live beneficial microorganisms, mostly *Lactobacillus*, *Bifidobacterium*, *Saccharomyces*, *Streptococcus*, *Enterococcus*, *Escherichia*, and *Bacillus* [12]. In men, some studies have indicated that the oral intake of probiotics can improve sperm quality and help reduce damage factors like ROS (reactive oxygen species) levels and DNA fragmentation. The intake of *Lactobacillus rhamnosus CECT8361* and *Bifidobacterium longum CECT7347* resulted in sperm motility improvement of asthenozoospermic patients [96]; additionally, the administration of different *Lactobacillus*, *Bifidobacterium*, and *Streptococcus* species resulted in an increase in a sperm concentration and motility [97]. The effects that these probiotics may have in the seminal microbiota still needs to be studied, although the use of *Lactibacillus* as a countering mechanism for the negative effect of *Prevotella*, *Pseudomonas*, and *E. coli* has been already suggested [57,78]. Regarding prebiotics, a study with oligoasthenoteratozoospermic individuals showed that the prebiotic Flortec, containing *Lactobacillus paracasei* 86 B21060 among other elements, improved sperm count, motility, and morphology after six months of daily intake [98].

## 5. Technical Considerations in the Study of the Seminal Microbiome

Given the fact that seminal microbiota—and microbiota in general—has a heterogeneous and changing nature, designing a microbial characterization study is a challenging goal since technical variation could contribute to bias in this profiling. These potential sources of variation may include specimen inclusion and exclusion criteria, processing of the samples, accidental contamination, the selected biological fraction, and the chosen technology or platform.

Most of the studies published so far have used whole ejaculate samples for the analysis, regardless of the profiling strategy. Nevertheless, several authors include a centrifugation or wash step prior to the molecular analysis with the cellular fraction. This is frequent for the study of viruses in sperm cells [47,67,82] but can also be conducted in bacteria profiling methods [31,32,33,56,57,88]. Štšepetova et al. studied the bacterial populations in raw ejaculates and processed/washed sperm samples, finding remarkable differences between them [28], indicating potential differences in the microbiota of seminal fluid and associated with cell fraction.

This same work by Štšepetova et al. showed the risk of microbial contamination during the handling and processing of semen samples, in this case for in vitro fertilization (IVF). They compared the microbiota profiling of sperm samples before and after incubation, finding that the incubated samples displayed a more diverse composition of bacteria. They also studied IVF culture media and found that they were not sterile either. In all cases, *Proteobacteria* was the predominant genus [28]. Regarding contamination during molecular characterization protocols, the presence of diverse bacterial species has been associated with certain techniques, like the background apparition of several species of *Proteobacteria*, *Actinobacteria*, *Firmicutes*, *Bacteroidetes*, *Deinococcus-Thermus*, and *Acidobacteria* in sequence-based microbiome analyses [99]. Some authors take into account the potential contaminating nature of these species when interpreting their microbiota results [31].

Another source of variability is the selected method for microbial detection, which also implies an evolution of the obtained results over the years along with the development of novel profiling technologies. The first and most traditional studies of seminal bacteria in the 1990s were based on the culture of seminal samples in agar plates with selective media in order to grow aerobe and anaerobe colonies that were later identified by subculturing, colony morphology analysis, enzymatic testing, etc. By this method, it was frequent to identify genus like *Ureaplasma*, *Mycoplasma*, *Escherichia*, *Corynebacterium*, *Gardnerella*, *Chlamydia*, *Staphylococcus*, *Streptococcus*, *Peptococcus*, *Lactobacillus*, *Enterobacter*, *Klebsiella*, *Haemophilus*, *Morganella*, or *Citrobacter* [8,19,23,58,83]. Nowadays, culture approaches constitute a basic technique still in use that is usually combined with PCR or qPCR. This allows us to verify the results obtained or to detect certain species with unfavorable cultivable characteristics, like some *Ureaplasma*, *Mycoplasma*, *Trichomonas* and *Gardnerella* species [53,75].

In fact, the use of PCR and qPCR started to gain popularity in the 2000s’ decade, which were frequently used for the detection of *Mycoplasma*, *Ureaplasma* and *Gardnerella* [14,55,56,80], although other studies also included *Chlamydia* and *Neisseria* [56,80] and other species of bacteria and viruses to the panel like *HPV*, *Enterococcus*, *Streptococcus*, *Staphylococcus*, *Escherichia*, *Pseudomonas*, *Klebsiella*, and *Lactobacillus* [34]. This technique offered a more sensitive detection of different bacterial entities at a molecular level in comparison with culture, as well as a quantification of the sample in the case of using qPCR. Nevertheless, the need for a previous selection of the species of interest to be included in the analysis supposes a serious handicap.

This limitation was overcome with the incorporation of next-generation sequencing (NGS) to the microbiome research field. This technology is usually based on the sequencing of certain hypervariable regions of the 16S rRNA gene of bacteria, which allows the distinction of operational taxonomic units (OTUs) at species level. As metagenomics technology, this approach provides a massive molecular characterization and quantification without a previous selection of species and offers wide possibilities of subsequent bioinformatics analyses, like taxonomic diversity studies (alpha and beta diversity) or clustering. The characterization of semen microbiota through 16S rRNA sequencing has broadened the range of bacterial elements discovered and shed light on aspects like their potential association with health and fertility [9,25,26,34,79,87,89] or their possible inter-relations [22,57]. In addition, other NGS strategies have been applied in the study of seminal microbiota. Some authors have performed bulk RNA-seq over sperm samples and used the reads that were not aligned to the human genome to perform a transcriptome characterization of bacteria and viruses altogether [33,39,88]. Lundy et al. also combined the 16S rRNA sequencing strategy with shotgun metagenomics [13], and Garcia-Segura et al. performed a bacterial profiling full-length 16S rRNA sequencing [21,24].

In particular, in this last work, the authors studied the differences introduced in the results by the sequencing platform during NGS analysis of seminal plasma microbiota. They performed a parallel full-length 16S rRNA sequencing on Illumina’s MiSeq sequencing platform (widely used) and MinION platform from Oxford Nanopore. They found that the platform factor produced no major effects in detection at the phylum level, but they had an influence in the detection of genus, and in the relative abundance of bacteria. They suggested that there are several elements of the NGS analyses that may introduce variability in the results, like bacterial DNA extraction methodology, the hypervariable regions of 16S rRNA gene of choice, and the bioinformatic pipeline, and noted the importance of conducting multi-platform studies [24].

## 6. Discussion and Future Directions

**Figure 3:**
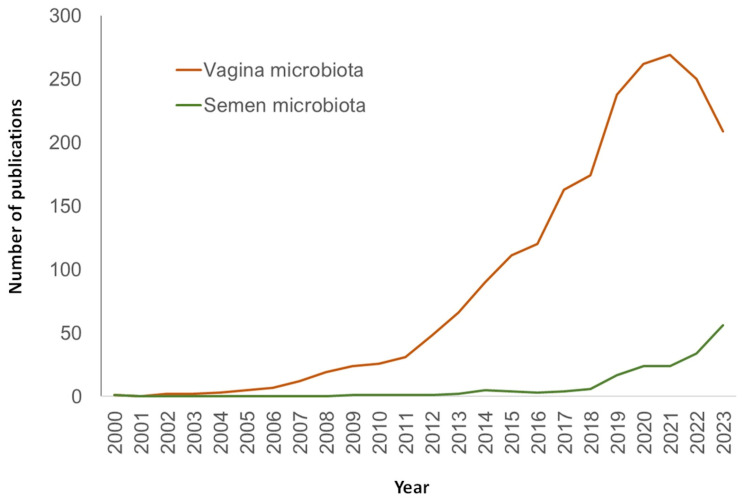
Number of publications in PubMed (URL [accessed on 15 January 2024] https://pubmed.ncbi.nlm.nih.gov/) per year and search query.

Over the years, the research of semen microbiome and its association with fertility has broadened through an increasing interest on the physiological role of microbiota and the use of NGS technologies. This has provided unprecedented information about the nature, characteristics, and species that inhabit the seminal fluid. In spite of this, the field of human seminal microbiome still presents obscure areas that highlight the need for deeper research. In fact, the number of publications dedicated to this topic, although increasing, is still considerably lower than studies dedicated to other microbiomes like vaginal ones (Figure 3).

One of the topics that are worth a deeper understanding is the specific pathways underlying the relation between certain bacterial/viral population and fertility disorders. As discussed above, many bacterial species and genus show an aberrant proliferation of scarcity associated with infertility or sperm parameters aberrations, suggesting an implication in processes related with fertility regulation. Besides this suggestion and its potential application in the field of fertility biomarkers, few specific pathways and dynamics have been elucidated. The interaction and equilibrium between microbiota and immune cells of mucosal tissues, and the consequences of dysbiosis, have been widely described in other human areas like the gut [1] or the female reproductive tract [100], suggesting similar mechanisms that would be reflected in the seminal microbiome. Untangling the key roles of bacteria in male fertility would help to understand the dynamics behind microbiota, semen quality and fertility itself.

Another topic of increasing interest is the influence of external factors over the seminal microbiome composition. Environmental disruptors like microplastics, air pollution, and other factors like stress or life habits are becoming increasingly present nowadays. In particular, human exposure and intake of micro- and nanoplastics, as well as the accumulation and effect on the organism, is being researched by many authors [101]. Different studies have detected consequences in the microbiota of human gut [102], and a recent study has evidenced the presence of microplastics in human semen [103]. The alteration of the human seminal microbiota after exposition to these factors is expectable, and it is becoming a key aspect to be researched.

A deeper understanding of the microbiome dynamics in the semen fluid and the influence of disruption factors would help to establish novel treatments or approaches for improving the microbiota and the fertility status. Given the potential of ingesting probiotics for improving seminal quality, they constitute a promising lead [104]. The application or combination with prebiotics for beneficial species and their effect on human male fertility still needs research and validation.

In order to design applications for the discoveries in the area, a solid research background needs to be reached. As hinted before, there is a general lack of consensus in the methodology of microbiome profiling studies, which often introduce bias in the results. This includes steps that are common in all microbiome studies (inclusion criteria, samples handling and contaminations, nucleotide extraction method, profiling technology, bioinformatics pipeline…), and others that are exclusive to semen analysis (the conditions of the sample obtaining, storage, optional separation of cell fraction from fluid…). Some initiatives have emerged as a response for this need in order to prove the magnitude of the effect of methodological choices over results and help establishing guidelines for correctly communicating the possible variation sources. This is the case of the National Institutes of Health (NIH) Human Microbiome Project, which was created “with the mission of generating resources that would enable the comprehensive characterization of the human microbiome and analysis of its role in human health and disease” [105]. This entity provides data, guidelines, and protocols for microbiome studies, encouraging researchers to reach a consensus. Additionally, the Microbiome Quality Control (MBQC) project consortium was founded with intent “to improve the state-of-the-science in microbial community sample collection, DNA extraction, sequencing, bioinformatics, and analyses, while promoting open sharing of standard operating procedures and best practices throughout the field” [106,107].

In conclusion, there is still much to learn about the composition, role, pathways, treatment, and potential applications of the human seminal microbiome, as well as its influence on fertility and health. Nevertheless, novel metagenomics tools are opening doors to new possibilities. In spite of the heterogeneity in the results and the limitations of the methodological aspects, the technical improvement continues providing new insights into the most representative species and the potential influence of ambiance and lifestyle factors over seminal microbiota. It is expected that new studies, driven under a more consistent consensus, will continue shedding light over this area.

## Data Availability

No new data were created.
